# How Participatory Music Engagement Supports Mental Well-being: A Meta-Ethnography

**DOI:** 10.1177/1049732320944142

**Published:** 2020-08-05

**Authors:** Rosie Perkins, Adele Mason-Bertrand, Daisy Fancourt, Louise Baxter, Aaron Williamon

**Affiliations:** 1 Imperial College London, London, United Kingdom; 2 Royal College of Music, London, United Kingdom; 3 University College London, London, United Kingdom

**Keywords:** mental health, mental well-being, meta-ethnography, music, participatory, qualitative, qualitative synthesis, well-being

## Abstract

Participatory music engagement has the capacity to support well-being. Yet, there is little research that has scrutinized the processes through which music has an effect. In this meta-ethnography [PROSPERO CRD42019130164], we conducted a systematic search of 19 electronic databases and a critical appraisal to identify 46 qualitative studies reporting on participants’ subjective views of how participatory music engagement supports their mental well-being. Synthesis of first-order and second-order interpretations using thematic coding resulted in four third-order pathways that account for how participatory music engagement supports mental well-being: managing and expressing emotions, facilitating self-development, providing respite, and facilitating connections. Our interpretation suggests that people benefit from participatory music engagement by engaging with specific and multiple processes that meet their individual needs and circumstances. These findings inform research directions within the field of music and well-being, as well as guiding the development and delivery of future music interventions.

## Introduction

With the advancement of the field of positive psychology, mental health is no longer classified as the absence of negative symptoms (Seligman & Csikszentmihalyi, 2014). Indeed, Keyes (2005) argues that mental health must be viewed as a “complete state” (p. 546): a separate, albeit linked, construct distinct from mental illness. Complete mental health can be thought of as comprising three components: emotional well-being, psychological well-being, and social well-being (Keyes, 2002). *Emotional well-being* includes the presence or absence of both positive and negative affect (Keyes, 2002) and has links with what others describe as hedonic well-being (the extent to which individuals experience happiness) and evaluative well-being (life satisfaction; Steptoe et al., 2015). *Psychological well-being* is defined by Ryff (2017) as the realization of human potential with six theoretically underpinned facets: purpose in life, self-acceptance, environmental mastery, positive relationships, autonomy, and personal growth. Recognizing the importance of social context in well-being, *social well-being* is defined by Keyes (1998) as including social integration, social contribution, social coherence, social actualization, and social acceptance. Both psychological and social well-being contribute to the idea of eudaemonic well-being, or “living life in a full and deeply satisfying way” (Deci & Ryan, 2008, p. 1). While hedonic and eudaemonic perspectives on well-being emerged separately (Ryan & Deci, 2001), well-being can be considered as one overarching concept that includes hedonia alongside multiple aspects of eudaemonia (Disabato et al., 2016). Mental well-being can, therefore, be understood as an absence or reduction of mental illness or symptoms as well as a presence or increase in emotional, psychosocial, and/or social well-being.

Writing on eudaemonic well-being, Ryff (2017) points to the need to understand better how the arts promote well-being across society. Indeed, there is an established body of research that links engagement with music to enhanced mental well-being (Fancourt & Finn, 2019). A recent systematic review, specifically focused on music and well-being, concluded that there is reliable evidence for positive effects of music and singing on hedonic and eudaemonic well-being in adults, including associations between music and reduced anxiety in young adults, enhanced mood and purpose in adults, and well-being, quality of life, self-awareness, and coping among people with health conditions (Daykin et al., 2018). Additional reviews, while acknowledging methodological and conceptual limitations in the evidence base, have demonstrated preliminary evidence for group singing supporting health-related quality of life, anxiety, depression, and mood in adults with chronic health problems (Reagon, Gale, Enright et al., 2016); music interventions benefiting anxiety and quality of life in people with cancer (Bradt et al., 2016); and rhythm-centered music-making improving anxiety, depression, anger, stress, mood, self-esteem, and motivation among diverse participant groups (Yap et al., 2017).

Despite this emerging evidence base, Daykin et al. (2018) point to a lack of research that focuses on the processes of change that account for music’s positive effects. In fact, in health research more widely, there is acknowledgment that pre/posttest designs such as randomized controlled trials (RCTs) need to be “combin[ed] with other methods, including conceptual and theoretical development, to discover not only ‘what works,’ but ‘why things work’” (Deaton & Cartwright, 2018, p. 2). This point has been made in relation to music by DeNora and Ansdell (2014), who argue that RCTs leave the music itself hidden and, therefore, the processes through which music has an effect also hidden. With limited knowledge of the processes underpinning change, the field is limited in its ability to scale up and operationalize music as a mental well-being intervention.

In response, quantitative studies have explored biological (Finn & Fancourt, 2018), psychoneuroimmunological (Fancourt et al., 2014), cognitive-emotional (Juslin & Västfjäll, 2008), psychophysiological (Ellis & Thayer, 2010), social-evolutionary (Tarr et al., 2014), and neurological (Koelsch, 2014) mechanisms that account for music’s effects. Yet, the experienced subjective processes of change for individuals remain far less known, despite some recent qualitative studies shedding light on how different forms of participatory music engagement can support different groups (Perkins et al., 2016, 2018; Warran et al., 2019). Other authors reflect on processes of change theoretically (e.g., MacDonald et al., 2012), and DeNora (2000) takes a sociological perspective to suggest that music has affordances (such as moods or messages) that people use in their “world-building” (p. 44). Music’s affordances are constructed within social contexts, such as new experiences, broadened horizons, and supportive interactions in youth justice settings (Daykin et al., 2017). This means that qualitative studies focusing on processes of change tend to be, by necessity, specific to particular contexts and groups of people, as well as frequently relatively small-scale. A higher level interpretation of the experienced subjective processes underpinning how participatory music engagement supports mental well-being, across different contexts, remains lacking.

### Meta-Ethnography

There has been a dramatic increase in the number of studies using qualitative synthesis to explore research in both health care and the creative arts therapy fields, aiming to make qualitative findings of more clinical relevance (Edwards & Kaimal, 2016). In particular, meta-ethnography is reported as the most commonly utilized method for qualitative health care research synthesis, being applied to a variety of health-related studies (Toye et al., 2014). Meta-ethnography was first established by Noblit and Hare (1988) as a means of synthesizing ethnographies conducted in educational contexts. Rather than providing an aggregated synthesis of qualitative studies, Noblit and Hare (1988) conceived of meta-ethnography as an interpretive endeavor, intended to construct new interpretations across a set of studies. Meta-ethnography in its original form comprised seven steps: (a) getting started, (b) deciding what is relevant to the initial interest, (c) reading the studies, (d) determining how the studies are related, (e) translating studies into one another, (f) synthesizing translations, and (g) expressing the synthesis (Atkins et al., 2008; Noblit and Hare, 1988). Through this process, and particularly that of translating studies into one another—where data across articles are compared in order to explain phenomena—“meta-ethnography is suited to producing a new interpretation, model or theory, which goes beyond the findings of the individual studies synthesised” (France et al., 2019, p. 2).

There are many examples of meta-ethnography in health care contexts, including in relation to the experience of Parkinson’s disease (Soundy et al., 2014) and women’s perceptions and experiences of traumatic births (Elmir et al., 2010). In mental health care, meta-ethnography has been used to investigate topics including mental health among Black men (Watkins et al., 2010) and the opportunities and obstacles employees with common mental disorders experience when returning to work (Andersen et al., 2012). While few meta-ethnographies have been conducted in relation to the arts and mental well-being specifically, O’Callaghan et al. (2016) investigated the relevance of music for people affected by cancer. Based on five studies, their review demonstrated the different ways, both positive and negative, that music was used by participants, recommending that music therapists are well placed to support people to make use of music in ways that benefit their health.

As these studies have demonstrated, meta-ethnography is applicable to health and mental health contexts. It provides a useful tool for synthesizing information from both researchers’ and participants’ perspectives (Hannes & Macaitis, 2012) and is well suited for the investigation of underexplored topics, combining research to extend existing knowledge (O’Callaghan et al., 2016). It also facilitates consideration of social phenomena in new ways, anchoring studies together to fill in knowledge gaps to generate original theory (Watkins et al., 2010). It therefore lends itself particularly well to advancing our understanding of *how* music is reported by participants to support mental well-being, an area that has so far received relatively little academic attention and that relies largely on qualitative, idiosyncratic data. Meta-ethnography offers a form of synthesis that brings together a body of qualitative research to make it stronger than the sum of its parts through a systematic process that generates new theoretical insight (Brookfield et al., 2019; France et al., 2019). In this article, we report on a meta-ethnography designed to answer the following research question:

**Research Question:** How does participatory music engagement support mental well-being?

## Method

To focus the meta-ethnography, we concentrated specifically on participatory music engagement (music-making) and excluded studies relating to music therapy or music-listening. Participatory music engagement is defined as music that is actively made by the participant, including singing, and is not limited by musical genre (e.g., popular, classical, or jazz). The main outcome under investigation was participant accounts of how participatory music engagement supported their mental well-being. The review protocol was registered with PROSPERO [CRD42019130164] and the meta-ethnography followed the seven steps identified by Noblit and Hare (1988), organized into three main sections: (a) systematic search, (b) critical appraisal, and (c) synthesis (Malpass et al., 2009).

### Systematic Search

A systematic search of the following major electronic databases was conducted: Cumulative Index of Nursing and Allied Health Literature (CINAHL), PubMed, ProQuest, Web of Science, WorldCat, WorldWideScience, SpringerLink, PubPsych, JSTOR, The Cochrane Library, OVID, PsyArticles, Embase, Global Health, PsycInfo, Education Resources Information Center (ERIC), Scopus, The Campbell Collaboration, and Joanna Briggs Institute. The search strategy included terms relating to or describing how (a) participatory music engagement supports (b) mental well-being through the use of (c) qualitative data (see supplemental Table 1). As many of the databases accepted different search strings, the search strategy was adjusted to each database as needed to maximize the volume of applicable results retrieved for each.

Two authors independently searched the included databases using the agreed search terms, dividing the available databases between them. The returned articles’ titles and abstracts were used to identify studies that potentially met the inclusion criteria: primary qualitative studies, including those using multi-strategy approaches, reporting on participants’ subjective views on how participatory music engagement supported mental well-being. “How” referred to the attributes of participatory music engagement that participants reported to be beneficial, rather than outlining the benefits per se that this activity was seen to bestow. Studies were excluded if they were quantitative; did not report on participants’ subjective views on how participatory music engagement supported their positive mental well-being (including studies only focused on outcomes); focused on clinical music therapy, physical, or physiological change; reported only on nonparticipatory music engagement (e.g., music-listening), or included participatory music engagement but only as a minor and indistinguishable component of another practice (e.g., singing as one part of a larger cross-arts intervention); did not have ethical approval or ethical process; included participants aged 16 years and below; were published before 1980; were not written in English; or were not peer-reviewed. Duplicates were removed and the full text of the potentially eligible studies were retrieved and independently assessed for inclusion by two authors. The searches were carried out between March and July 2019, with the last search taking place on July 1, 2019.

### Critical Appraisal

There is significant debate surrounding how to evaluate qualitative research (Barbour, 2001). Following other meta-ethnographies, to assess the full texts for potential inclusion, we made use of a checklist based on that reported by Atkins et al. (2008), although we modified it to acknowledge France et al.’s (2019) point that rich primary data are essential to a meta-ethnography, allowing for further interpretation within the meta-ethnography process. This was an important additional checkpoint as there are few purely ethnographic studies relevant to the study’s aims. Therefore, a wider range of qualitative methodologies and methods were included, but only if the primary data were considered rich enough to form the basis for our interpretive analysis. Our criteria for establishing this, agreed by two authors, were (a) sufficient amounts of primary qualitative data, meaning that articles with few or no first-level quotes were not included, *and* (b) inclusion of author interpretation, meaning that articles listing or describing primary data without interpretation or discussion were also not included. Failure of this additional check did not infer that articles contained low-quality research.

The first two questions within the quality checklist were used as screening items: “Is the study qualitative research” and “Does the study include rich qualitative data?” If articles did not achieve a “yes” for both items, they were excluded as unsuitable for the purposes of this review. Thereafter, agreeing with Barbour (2001) that “overzealous” application of checklists does not ensure rigor, articles were included if they achieved “somewhat” or “yes” on each of the other quality check items. All selected articles were reviewed for quality by two authors. Any disputes over inclusion were discussed and, although reference to a third author was planned for the event of conflicting opinions, full consensus was reached over which articles to include. The included articles’ reference lists were also searched for potential articles for inclusion and one new article was identified. The article selection process is summarized in supplemental Figure 1.

### Synthesis

Two authors independently identified and extracted quotes (first-level interpretations) that outlined how participants felt that participatory music supported their mental well-being, along with corresponding author interpretations and discussions (second-level interpretations), with all conflicts of opinion solved through discussion. The same authors read and discussed the resulting data, using second-level interpretations as the main unit of analysis, given the importance in meta-ethnography of reinterpreting existing author interpretations (France et al., 2016). All second-level interpretations, supported by their corresponding quotes, were grouped into codes in a process of reciprocal translation (comparing data across articles to explain phenomena), which involved grouping similar meanings across the different studies. The relationships between these broad codes were then examined, and related codes were grouped into categories that formed our third-order constructs (Brookfield et al., 2019).

To ensure the validity of the analysis, codes and categories were cross-checked independently by a third author. The third reviewer largely agreed with the coding and categorization but suggested some alterations. The codes and categories were therefore revised before being resubmitted to the third reviewer who confirmed the altered categorization. Once the coding was approved, a final analysis session was conducted with a wider group of researchers from the lead research group to complete a final sense-check and cross-check of second- and third-level constructs. Whereas the methodology is presented here in a linear manner, the synthesis of the articles was a reciprocal process, with our codes evolving as new categories emerged from our analysis and interpretations.

Mason-Bertrand and Baxter conducted the systematic search. Mason-Bertrand and Perkins conducted the full-text inclusion and quality checks, data extraction, and synthesis with Williamon. Fancourt conducted the cross-check. Perkins led study design and write up, to which all authors contributed.

## Results

A total of 46 articles were selected for the meta-ethnography, as summarized in supplemental Table 2, with a total sample size of at least 2,164 participants. The precise total is unknown as some articles that employed a multi-strategy approach did not outline the specific number of participants within the qualitative components of their studies. The authors ensured that participants were not counted more than once when participant samples were used across multiple included articles. Some of the included articles also discussed participants who engaged in music-listening as well as those who were involved with music-making, however, only data relating to music-making were extracted from these articles and the sample size only includes those participants who specifically engaged in music-making [1, 2, 38] (note: these numbers in the square brackets at the end of the citation refer to the references with the corresponding number).

The included articles were published between 2002 and 2019 and were conducted in a range of countries, with some studies pertaining to more than one country: Sweden (*n* = 2), United Kingdom (*n* = 22), Canada (*n* = 3), Norway (*n* = 2), Rwanda (*n* = 1), Australia (*n* = 10), United States (*n* = 4), Germany (*n* = 2), South Africa (*n* = 2), China (*n* = 1), and New Zealand (*n* = 1). Methods used included qualitative interviews (*n* = 35), focus groups (*n* = 18), qualitative questionnaires (*n* = 8), and observations (*n* = 4), with some articles employing mixed methods. Participants included older adults (*n* = 4), people experiencing chronic health conditions (*n* = 7), people with intellectual disabilities (*n* = 1), people with physical disabilities (*n* = 1), people with mental illness (*n* = 7), carers (*n* = 4), people experiencing homelessness (*n* = 2), people with dementia (*n* = 1), pregnant women (*n* = 2), war orphans (*n* = 1), health care professionals (*n* = 5), middle-class people (*n* = 1), music facilitators (*n* = 3), new mothers (*n* = 1), migrants (*n* = 4), veterans (*n* = 1), and people experiencing bereavement (*n* = 2). One article [5] compared the experiences of music-making across two different participant groups (middle-class people and people experiencing homelessness). The articles contained different types of music participation and some articles featured more than one method of music-making: group singing (*n* = 34), individual singing (*n* = 4), group lullaby singing classes (*n* = 2), individual keyboard lessons (*n* = 1), group keyboard lessons (*n* = 1), individual recorder lessons (*n* = 1), group recorder lessons (*n* = 1), individual guitar (*n* = 3), group guitar (*n* = 1), piano (*n* = 3), song writing (*n* = 2), orchestral playing (*n* = 1), group drumming (*n* = 5), playing in a band (*n* = 1), percussion (*n* = 3), ukulele (*n* = 1), steel pans (*n* = 1), and playing an unspecified musical instrument (*n* = 4).

The process of synthesis resulted in 22 codes (processes), grouped into four overarching categories: managing and expressing emotions, facilitating self-development, providing a form of respite, and facilitating connections.

### Managing and Expressing Emotions

Synthesis revealed that music participation provides a means of emotional connection, expression, management, and release, as well as eliciting uplifting emotions and relaxation. This category comprises six processes, as summarized in Table 1.

**Table 1.** Summary Descriptions for Category 1: Managing and Expressing Emotions.

Process	Description	Articles
1.1. Connecting to and expressing deep-seated emotions	Music participation allows people to explore and express deep-seated emotions	4, 6, 8, 22, 29, 31, 38, 39, 45
1.2. Coping with emotions	Music participation helps people to cope with negative emotions	2, 4, 5, 6, 8, 17, 21, 22, 23, 29, 34, 38, 44
1.3. Eliciting uplifting emotions	Music participation elicits positive emotions	1, 2, 3, 4, 5, 6, 7, 8, 9, 12, 13, 14, 17, 19, 20, 21, 22, 24, 25, 26, 27, 28, 29, 30, 32, 34, 35, 38, 39, 41, 43, 44, 45, 46
1.4. Facilitating catharsis	Music participation allows people to release negative emotions	4, 5, 6, 15, 17, 18, 22, 25, 27, 29, 46
1.5. Perceiving the benefits of music	People *believe* that music participation supports positive emotions	4, 5, 6, 7, 9, 15, 17, 20, 21, 22, 23, 25, 27, 33, 37, 38, 41, 43, 44, 45
1.6. Providing relaxation	Music participation makes people feel more relaxed	5, 6, 7, 8, 12, 17, 24, 34, 35, 41, 45

#### Connecting to and expressing deep-seated emotions

Music participation provides a way for people to connect with and access emotions. This is particularly the case for deep-seated or even nonconscious emotions that may be otherwise difficult to access, negative, or linked with challenging circumstances:

Singing was spoken about as a way of accessing and expressing difficult emotions, and was seen as a safe, controlled mode of expression which helped to connect to difficult emotions without feeling overwhelmed. This was especially important for those who found it difficult, who were not aware of their emotions in their everyday lives. [Article 45, p. 50]

Participants expressed that the emotional rollercoaster of caring for an unwell relative and grief were intimately intertwined with their own sense of wellbeing and health. The participants described how singing enabled them to get in touch with their feelings and offered an opportunity to explore emotions, and this was key to their wellbeing. [Article 6, p. 5]

In some of the articles, music was explicitly mentioned as a means of expression that is an alternative to language [8, 29, 31, 39].

#### Coping with emotions

Music participation is a way for people to cope with negative emotions associated with adverse life events:

The activity of singing itself helped the participants overcome difficulties in their lives and manage their emotions while feeling valued and grateful. [Article 22, p. 184]

Singing in the choir was portrayed by some as a meditation, which enabled them to cope when they felt stressed. [Article 6, p. 6]

The way in which music was used to cope was explicitly linked with the specific circumstances of participants; for example, in coping with grief [4, 6, 23, 38, 44] or illness [2, 6, 23, 38].

#### Eliciting uplifting emotions

The act of engaging in music also elicits positive, uplifting emotions among participants:

[The drumming group] produced feelings of euphoria, vitality, and well-being. These positive mood states were enduring and authentic. Participants reported that the drumming group could change negative mood states to positive, describing it as “uplifting.” This effect extended to both their working and personal lives. [Article 29, p. 446]

This music, along with other “cheerful music,” made Christine feel as though nothing could stand in the way of her happiness. [Article 2, p. 159]

The use of words, such as fun [17, 21, 26, 39, 46], uplifting [19, 21, 24, 29, 34, 43, 46], positive emotions [3, 21, 27, 34, 45], joy [3, 4, 6, 21, 27], and enjoyment [5, 8, 17, 25, 30, 35, 39, 46] explain the various ways in which music is perceived to elicit positive emotions.

#### Facilitating catharsis

In addition to allowing people to *connect* with and *cope* with emotions, music participation also allows participants to *release* negative emotions:

For almost 25 years Patrice has been haunted with feelings of regret. He believed that he had disappointed his Mother by not pursuing the career path she had wanted him to follow. Patrice maintains that singing in the choir has provided him with a cathartic experience that has released him from the guilt he carried with him for so many years. This incident . . . does not seem to be a habitual behaviour used to deal with periodic depressive episodes, but rather a single occurrence that allowed Patrice to move forward. [Article 4, pp. 237–238]

The articles refer to catharsis either in its own terms, or with reference to the “release” or “ridding” of negative emotions [4, 5, 6, 15, 17, 27, 46].

#### Perceiving the benefits of music

An important aspect of the way in which music supports mental well-being is that the participants *believe* it to have an effect. Across numerous articles, participants state their perception that music is beneficial, both mentally and physically:

The participants in the current study also had strong feelings about the beneficial aspects of music making, with social and musical benefits being the most prevalent response. [Article 37, p. 9]

The participants also reported important benefits on their overall physical condition and posture. Posture is an important aspect of singing; the teacher therefore taught the participants exercises for the neck and upper body. Some noticed that they were standing straighter than before. Others noticed a difference in their energy levels. [Article 22, p. 184]

Participants refer to the “therapeutic properties” of music [4, 17, 43] or its potential to “heal” [4, 15], with some also referring to music being a perceived alternative to medication or with the ability to reduce physical symptoms [6, 7, 17, 38 43].

#### Providing relaxation

Finally, music participation also facilitates a sense of calm and relaxation, which the participants link with enhanced mental well-being:

The aesthetic beauty of the music and lyrics acted as a balm and participants spoke of feeling peaceful and relaxed as the music soothed their concerns. [Article 8, p. 324]

Duncan expressed how his mental health led to him losing his job and becoming depressed. The choir provides a relaxed environment, allowing an improvement in his psychological health and has eased his feelings of depression. [Article 35, p. 35]

This code is also linked with *coping with emotions*, allowing participants to reduce stress through relaxation and soothing [6, 7, 8, 12, 35].

### Facilitating Self-Development

Synthesis revealed that music participation provides a sense of purpose, providing opportunities for participants to develop skills and supporting their accomplishment, agency, self-confidence, and identity formation. This category comprises six processes as summarized in Table 2.

**Table 2.** Summary Descriptions for Category 2: Facilitating Self-Development.

Process	Description	Articles
2.1. Developing skills	Music participation leads to the development of new skills	4, 5, 9, 17, 22, 23, 25, 28, 29, 32, 33, 36, 44, 46
2.2. Facilitating accomplishment	Music participation requires effort, which grants a sense of achievement	3, 4, 5, 8, 9, 14, 17, 22, 23, 26, 27, 28, 29, 32, 34, 44, 45
2.3. Giving a sense of purpose	Music participation gives people a sense of meaning, hope, and resilience as well as structure in life	3, 5, 9, 12, 17, 22, 23, 24, 25, 27, 28, 30, 32, 34, 35, 38 42, 44, 45
2.4. Promoting agency	Music participation supports people to take the initiative and engage in new activities	3, 17, 22, 27, 35, 39, 41, 42, 43, 45
2.5. Promoting self-confidence	Music participation helps to build confidence, particularly during times of challenge	3, 5, 6, 7, 8, 12,14, 17, 22, 24, 25, 26, 27, 28, 31, 35, 39, 40, 41, 44, 45, 46
2.6. Supporting identity formation	Music participation supports self-discovery and identity formation and expression	1, 2, 3, 5, 6, 12, 15, 17, 22, 29, 32, 33, 34, 36, 43, 45, 46

#### Developing skills

Music participation provides a way for people to develop new skills:

As they engaged in a challenging activity, the participants developed and honed physical and cognitive skills on a daily basis. The activity of singing, the whole learning process, the voice training, and the mind and body exercises all had a definite impact on their personal abilities, including cognitive skills. [Article 22, p. 183]

The Choir appears to provide a focus for mental energy. The motivation and discipline required to attend rehearsals, learn the repertoire and perform for the public result in increased skill. Increased skill permits movement to new levels of understanding and promotes further knowledge acquisition and skill development. [Article 4, pp. 243–244]

Specific skills referenced include general skill development [17, 22, 23], cognitive skill development [9, 22, 25, 44], and musical skill development [4, 5, 28, 29, 32, 33, 36, 46].

#### Facilitating accomplishment

Linked with skill development, music participation grants a sense of achievement:

There was a sense of enjoying the challenges which come from being part of a singing group. Singing was perceived as something difficult but rewarding, and participants emphasised the importance of having something in your life that you put effort into. [Article 45, p. 49]

The specificities of drumming that optimize the sense of accomplishment were also highlighted, especially the easiness in producing sound, regardless of one’s skills. [Article 3, p. 7]

Rather than being consumers of music—typically through listening or watching others—participants expressed satisfaction at their ability to be, themselves, the producer of music. [Article 32, p. 561]

Achievement is specifically linked to taking on and succeeding with a challenge [3, 22, 23, 26, 28, 29, 44] as well as being able to make and perform music [3, 5, 14, 17, 22, 27, 28, 32, 44].

#### Giving a sense of purpose

Music participation gives participants a sense of purpose in life:

The choir was considered by some as part of a personal journey in pursuit of meaning and purpose, and ultimately personal growth . . . Several interviewees stated that the choir also allowed them to discover a deeper purpose in life. [Article 17, p. 128]

In particular, the singers in the Group A, who regularly visited nursing homes to provide musical performances, explained how these special visits provided them with something to work towards as well as a meaning, and that they felt they provided a meaningful service to the community. In addition to finding meaning in their participation by providing a service to the community, some participants also found a personal purpose and meaning to their attendance. [Article 23, p. 200]

Purpose is also intertwined with meaning [3, 17, 23, 27], resilience especially in the face of illness [17, 42, 45], and hope [30, 35]. It is also linked with the structure that regular music-making can provide [9, 12, 22, 23, 32, 34, 45], giving participants routines and consistency.

#### Promoting agency

Music participation supports participants to take the initiative, to make plans, and to engage in new activities:

Enhanced motivation propelled some [participants] to make future plans, increasing their hope and optimism. The choir also appeared to “open doors” to discovery and pursuit of other opportunities by enhancing self-efficacy, as it enabled participants to recognise their own capacity to achieve, commit and self-motivate. This led to the development of new interests for some, including joining other social groups (Eddie) and volunteering (Mary). [Article 17, p. 128]

All participants reported enhanced proactive-ness and a greater ability to act on their own will and make free choices. Here, there were recurrent references to new behaviours denoting initiative. . . . the attendance of the sessions themselves was reported as a first step of intentionality and autonomy. [Article 3, p. 5]

Agency is explicitly linked with trying out or engaging with new activities beyond the music-making itself [3, 22, 35, 39, 41, 43] and to having a sense of control in life [3, 17, 22, 27, 42, 45].

#### Promoting self-confidence

Music participation helps to build confidence:

It seemed the choir gave confidence to participants in a number of ways, including defying negative associations of singing from childhood, giving general confidence and empowering participants at a time where confidence had been lost due to being affected by cancer. [Article 46, p. 4]

By regaining confidence in themselves, many testified how succeeding in the activity gave them hope and allowed them to gain optimism about their own future. They shared how they didn’t have the confidence to face and to adapt to everyday challenges in life before the beginning of the activity. [Article 22, p. 182]

For some of the participants, increased self-confidence is related to overcoming anxieties or previous negative associations with music [7, 22, 26, 46], the collective achievement of being part of a group [6, 41], and feeling accepted socially [3, 5, 40].

#### Supporting identity formation

Music participation supports participants to (re)connect with their sense of self:

All participants reflected on the choir’s role in developing the “self” and promoting internal changes. Interviewees presented this as a journey of self-discovery, beginning with the development of self-awareness and knowledge through members’ recognition of their abilities and limitations. This was followed by learning to accept one’s self which was facilitated by peer validation. [Article 17, p. 129]

Finding a voice was a subtheme that contributed to both the personal impact and social impact of the choir. People experiencing chronic mental health problems and disabilities often do not receive much attention by society but the choir was an opportunity to make themselves heard. [Article 12, p. 414]

This identity work operates on many levels, including supporting participants to reconnect or rediscover their identity [3, 5, 6, 17, 22, 32, 33, 34, 45, 46]. It also gives participants the opportunity to step outside of an ascribed identity or role [1, 15, 17, 29, 33, 36, 43], to be self-aware [3, 12, 17, 43], and to feel that their voice is being heard [5, 12].

### Providing Respite

Synthesis revealed that music participation creates a space of safety and me time, providing opportunities for distraction and absorption. This category comprises four processes as summarized in Table 3.

**Table 3.** Summary Descriptions for Category 3: Providing Respite.

Process	Description	Articles
3.1. Creating a safe space	Music participation provides a space of safety, both during and outside of structured engagement	3, 4, 5, 8, 11, 12, 17, 21, 22, 28, 29, 33, 34, 35, 39, 40, 45, 46
3.2. Creating me time	Music participation provides protected time for people, especially important for those with caring responsibilities	7, 13, 26, 34, 43, 45
3.3. Providing distraction	Music participation can distract from challenges or worries	1, 2, 4, 5, 7, 8, 9, 17, 19, 25, 26, 27, 29, 32, 38, 41, 45
3.4. Providing absorption	Music participation can be absorbing, allowing people to lose themselves	3, 4, 13, 18, 22, 25, 27, 28, 29, 32, 33, 34, 46

#### Creating a safe space

Music participation provides a space of safety, both during and outside of structured engagement:

This notion of safety also presents in the current study and Donna gives a moving account of her experience of caesarean section where trees blowing in the wind outside the operating room window triggered a visual image associated with one of the study lullabies. This image had the effect of reproducing feelings of relaxation and safety linked with singing the lullabies and Donna recalls feeling safe and confident that all would be well. [Article 8, p. 326]

The group emerged . . . as facilitating acceptance, with judgements put aside in favour of an inclusive, integrated environment. [Article 33, p. 10]

Safety relates to feelings of security and comfort [4, 5, 8, 11, 28, 29, 33, 34, 39, 45, 46], safe ways to connect with others [3, 4, 5, 12, 17, 35, 45, 46], as well as experiences of a nonjudgmental and accepting environment [3, 21, 22, 28, 33, 35, 40].

#### Creating me time

Music participation is time for oneself:

The singing sessions also emerged as a form of “me time,” where the mothers could do an activity for themselves. [Article 34, p. 6]

One caregiver experienced frustration in the role of caring, a sense of freeing from this frustration was found in the act of singing. [Article 43, p. 475]

In addition to finding “me time,” music participation facilitates a sense of freedom from caring responsibilities [7, 34, 43] as well as providing a way to remain anonymous [45].

#### Providing distraction

Music participation provides a distraction from day-to-day concerns as well as from adverse life circumstances:

The song lyrics Christine listened to or sang created an imaginary world in which she was no longer a middle-aged woman with breast cancer, but a cheerful young girl who had no problems and lived in total happiness. [Article 1, p. 236]

Singing was seen as a way of switching off and having a break from life’s troubles. [Article 45, p. 50]

Whereas music was most often used as a means to distract from worry, problems, or physical symptoms [1, 4, 5, 7, 8, 9, 19, 25, 26, 27, 29, 32, 38, 41, 45], it was also used the opposite way, to facilitate a connection to, and acceptance of, pain [17].

#### Providing absorption

Music participation can be absorbing, allowing participants to lose themselves:

For the mothers, singing appeared to offer an immersive experience that provided some relief from the practical and emotional concerns of early motherhood. [Article 34, p. 9]

There were accounts of moments of extreme involvement, denoting a sense of energized focus, suggesting experiences of flow. Participants highlighted the sessions as strongly immersive, frequently leading to a sense of getting taken away. [Article 3, p. 7]

The absorption is linked with grounding or spiritual experiences [25, 27, 33] as well as with being in the moment or in a flow state [3, 4, 13, 18, 22, 28, 29, 32, 34, 46].

### Facilitating Connections

Synthesis revealed that music participation facilitates connections with other people, with heritage, and with the past, providing opportunities to contribute to society, to feel togetherness and belonging, and to experience social support and enhanced social functioning. This category comprises six processes as summarized in Table 4.

**Table 4.** Summary Descriptions and Indicative Evidence for Category 4: Facilitating Connections.

Process	Description	Articles
4.1. Connecting through music	Music participation creates connections between people through the music itself	3, 4, 6, 7, 13, 21, 28, 29, 33, 34, 39, 40, 41, 43
4.2. Connecting to heritage and past	Music participation creates a sense of connection to heritage, and allows people to reminisce and feel connected to past events	7, 11, 19, 24, 25, 30, 34, 37, 42, 43
4.3. Creating opportunities to give and contribute	Music participation creates opportunities for people to contribute to society	3, 4, 5, 12, 13, 17, 20, 23, 25, 28, 31, 44, 45
4.4. Creating togetherness and belonging	Music participation provides a sense of fellowship, bringing together people with shared experiences as well as differences	3, 5, 6, 7, 9, 12, 13, 14, 16, 17, 19, 20, 21, 22, 23, 24, 25, 26, 28, 29, 30, 31, 33, 34, 35, 39, 40, 42, 43, 45, 46
4.5. Providing social support	Music participation provides social support and opportunities to support others	9, 14, 18, 22, 26, 30, 33, 34, 35, 41, 45, 46
4.6. Providing wider social benefits	Music participation supports social benefits beyond the immediate music-making context	3, 4, 7, 12, 21, 22, 28, 29, 32, 33, 39, 40, 43, 46

#### Connecting through music

Music participation creates connections between people through the act of making music:

It would appear that the vibrations and sounds of drumming are able to elicit a means of communication that is important to these participants because it relies on a different communication mechanism than verbal language. [Article 33, p. 7]

The participants attributed part of this connection to the nature of singing as an activity. Singing allowed co-construction of something beautiful with other people. Singing therefore facilitated a connection with others that the participants could then build on. [Article 28, p. 4]

Participatory music engagement is reported to support connection through the intensity of the shared experience of music-making and the arising group identity [3, 6, 7, 13, 21, 28, 29, 33, 34, 41, 43] as well as through supporting bonds and connections with others [3, 4, 6, 7, 13, 39, 40].

#### Connecting to heritage and past

Music participation provides a means for people to feel connected with their heritage and/or their past:

Being part of a choir and singing with others who share cultural and linguistic heritage is a positive and beneficial experience that offers older people opportunities for social bonding and belonging. [Article 42, p. 53]

Some participants reported a “carrying over” of singing ability from premorbid days that helped to tap into memories of previous times and identity. [Article 43, p. 475]

Connecting with heritage through music is seen by participants as supporting an enhanced sense of connection with others through shared cultural or historical contexts [19, 24, 34, 37, 42], while connecting with the past is viewed as a form of nostalgia and (re)connection with previous identities or memories [7, 11, 25, 30, 43].

#### Creating opportunities to give and contribute

Music participation provides a way to give something back or contribute to, and be part of, society:

Participating in concerts, especially when proceeds are used to assist other marginalized persons, allows some individuals to experience the sense that they are making a contribution. [Article 5, p. 282]

A sense of connection with the audience was another common theme. When asked about their favourite moment in the choir so far, a large number of people responded that is was during the performances and the warm reception from the audience. [Article 12, p. 415]

This process includes both the use of music as a means to support others and contribute [3, 5, 13, 17, 23, 25, 28, 31, 44, 45] as well as the use of music to feel connected with wider society [4, 5, 12, 20, 25, 45].

#### Creating togetherness and belonging

Music participation provides a sense of fellowship, bringing together people with shared experiences as well as differences:

Having a common cancer experience also provided participants with a connection. The choir gave them a sense of belonging and, for those who wished, an opportunity to share their experiences. This was deemed important because participants felt that people with no experience of cancer could not fully understand what they had been through. [Article 14, p. 7]

They [participants] also had to collaborate in a group despite differences. The participants mentioned that they all had different personalities and were proud to say that they cooperated through the difficulties they faced in the activity. [Article 22, p. 183]

This code was large, with belonging revolving around collective and shared experiences [6, 7, 14, 17, 24, 25, 28, 33, 43, 46], social bridging, or connecting with others despite differences [3, 5, 16, 21, 22, 26, 35, 40, 43, 45], belonging and togetherness [3, 5, 6, 9, 12, 13, 17, 19, 21, 23, 24, 26, 28, 29, 30, 33, 34, 35, 39, 40, 42, 43, 45, 46], and spirituality [12, 20, 31, 45].

#### Providing social support

Music participation provides social support and opportunities to support and care for others, as well as to be cared for:

One participant expressed how important the choir is to his recovery due to the support he feels from other choir members without feeling the stigma of mental illness. [Article 35, p. 34]

Whilst some participants stated that they had joined the choir specifically for support, others wished to provide support to other choir members. [Article 14, p. 7]

In some articles, social support is explicitly linked with reducing feelings of loneliness and/or isolation [9, 35, 41].

#### Providing wider social benefits

Music participation supports social networks and interactions both in and beyond the immediate music-making context:

Learning music appears to have facilitated meaningful and musical contact with someone beyond the music group. While group-learning settings may offer tangible opportunities to meet and socialize with new people, music-learning also appears to have the potential for facilitating new social interactions beyond the immediate music-learning context. [Article 32, p. 560]

The group acted as a source of new social contact, with participants regularly meeting before and after the sessions in the café attached to the venue. [Article 33, p. 10]

These social benefits include social interaction in or around music sessions [32, 33] and outside of music sessions [3, 4, 7, 12, 22, 28, 29, 32, 40, 43, 46], as well as supporting the development of social skills [3, 12, 21, 39].

## Discussion

In this article, we have synthesized the evidence base from data provided by more than 2,000 participants to identify four pathways by which music participation supports mental well-being. In discussing these pathways, three points require further elucidation. First, each pathway is itself comprised of multiple codes, capturing specific processes by which music participation supports mental well-being. Within the overarching pathway of connections, for example, some of the connections are facilitated in the moment by the immediate participatory music engagement (e.g., connecting through music, connecting to past and heritage, and creating togetherness and belonging), whereas others are facilitated by wider social connections that permeate out from the engagement (e.g., contributing to society, building social support, and experiencing wider social benefits). It is clear that each pathway encompasses distinct and yet overlapping processes. Second, and linked to the first point, neither specific processes nor overarching pathways are mutually exclusive. Participants benefit from music by engaging in multiple processes within *and* between the overarching pathways of managing and expressing emotions, facilitating self-development, providing respite, and facilitating connections. Indeed, as many of the processes and pathways are interwoven, during analysis, many of the second-level interpretations fell under more than one process. As a result, the second-level interpretations were placed under multiple codes, when applicable, to reflect the dynamic relationship identified between the processes.

Third, the *specificity* and *multiplicity* of the processes appear to be determined by the *individual needs and circumstances* of participants. While the pathways themselves may appear relatively generic, the ways that individuals engage in them are highly idiosyncratic. We see evidence of this, for example, in the process of “connecting to and expressing deep-seated emotions.” This ranged from pregnant women connecting through music with feelings of fear and love for their unborn child [8], to carers exploring through music the emotions involved in their caring responsibilities [3], to staff in a child and adolescent mental health unit expressing emotions through music connected with their work [29]. It seems that, while the meta-level pathways are consistent across the 46 articles included in this review, the needs of individuals—as shaped by their life circumstances and context—defines the ways in which they utilize the pathways to benefit their mental well-being. Indeed, such an interpretation is consistent with DeNora’s (2000) premise that music’s affordances are “constituted from within the circumstances of use” (p. 44), and that people actively appropriate music’s affordances according to the contexts in which they are situated (see also Daykin et al., 2017).

Following from this, while music was found to address the individual needs that arose from individual contexts, it is important to note that the effects of music-making varied according to the users’ cultural and social backgrounds. For example, two studies demonstrated that whereas middle-class choralists were aware of and restricted by musical elitism, choir members from marginalized backgrounds were not constrained by these notions [4, 5]. Therefore, despite participating in the same musical activity, music-making held different connotations for these two groups, which influenced how music-making addressed their needs. Furthermore, Process 4.2 of Table 4 highlights the importance of music being used to connect with people’s specific heritage and past. As music is argued to be “inescapably culturally situated” (Cross, 2012, p. 27), it appears that so too are its effects, with the social and cultural context of the music-maker impacting on the processes through which music supports their mental well-being.

**Figure 1.**
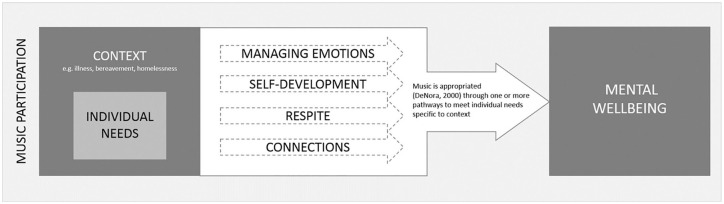
The pathways by which participatory music engagement supports mental well-being.

Figure 1 illustrates how music participation meets individual needs as they arise from particular contexts (e.g., illness [1, 2, 3, 12, 16, 17, 22, 28, 30, 39, 40, 41, 43, 46], bereavement [11, 36, 45, 46], and homelessness [4, 5]) through the four pathways identified in this study. These pathways, as discussed above, are multifaceted and also permeable; individuals report different benefits of music depending on what they need at any particular time. As DeNora (2000) suggests, it is perhaps this “interpretive flexibility” (p. 43) that makes music an effective intervention for mental well-being.

Scrutiny of Figure 1 reveals many overlaps with other theories of well-being and positive mental health. Indeed, music participation clearly meets the three components of well-being set out by Keyes (2002): *emotional well-being* (connecting to and expressing deep-seated emotions, coping with emotions, experiencing uplifting emotions, catharsis, relaxation, and perceiving positive benefits of music), *psychological well-being* (developing skills, experiencing accomplishment, purpose, agency, self-confidence, and building identity), and *social well-being* (connecting through music, connecting to heritage and past, opportunities to give and contribute, experiencing togetherness and belonging, building social support, and wider social benefits). The processes captured in the “respite” pathway, however, appear to fit less immediately within existing models of well-being, representing instead the need to step outside of day-to-day challenges or circumstances such as physical or mental illness. Music appears to meet the need to be transported somewhere else, to reclaim time for oneself in a safe environment, and to be distracted from daily concerns.

The work reported in this article is limited by challenges experienced during the review process. The first is perhaps a function of the available literature in the field, much of which tends to conflate *outcomes* with *processes*. This meant that data selection was at times challenging, as we had to take decisions about content that met our inclusion criteria: articles that referred to how participants reported that participatory music engagement supports their well-being, rather than the actual benefits themselves. In reality, many articles covered both, and we therefore took subjective decisions about which data to extract to meet our aims. Furthermore, the process of meta-ethnography is by definition interpretative, and thus the pathways outlined in this article are the result of an interpretative, subjective process of reanalyzing authors’ original interpretations. Indeed, we recognize that it is possible that these original interpretations, and the selection of primary data on which they were made, may reflect selection bias, and that they will have been constructed within different methodological and epistemological framings that we have not been able to fully capture when combining the secondary data. Although we have cross-referred across our author team, readers should remain mindful that this process has been, by necessity, interpretative on many levels.

In addition, we limited articles to those written in English, partly to create parity in how we engaged with the texts but also in recognition that our sample of 46 articles is already at the top end of what is recommended for meta-ethnography (Brookfield et al., 2019). Our conclusions are also limited by a dominance of group singing and choirs as the type of participatory music engagement reported in the existing literature (featuring in 34 articles out of 46), as well as a lack of information on the genre of music involved (only reported in 14 out of 46 articles).

The results are further limited by a focus on the positive effects of participation in music, mainly as a result of the inclusion criteria for article selection, but also reflecting a general lack of scrutiny of negative or unintended effects of music in much of the literature. However, we acknowledge that music-making is not always positive. Listening to or playing certain pieces of music associated with unpleasant events, for example, can heighten feelings of melancholy or exacerbate negative emotional states (Hays & Minichiello, 2005). In addition, whereas music-making has been seen to reduce levels of stress, performing music can also elicit negative emotions and heighten feelings of anxiety (Steptoe, 2001).

Furthermore, although this article has demonstrated how music-making may aid in building social capital, bridging differences between individuals and encouraging meaningful interactions, music-making can also heighten social inequalities. For example, Green (2012) argues that Western classical music is widely regarded as the most valuable genre for musical education, corresponding with middle- and upper-class tastes and ideology. Consequently, she suggested that whereas middle- and upper-class children were more likely to have the knowledge of classical music needed for a musical education, and the wealth to afford private tuition, working-class children lacked these resources and were therefore less likely to do well in music and more likely to stop pursuing music-making. Inequalities surrounding music-making can also apply to sex and ethnicity, with both female and ethnic minority composers, for example, being more likely to be excluded from the music curricula, which may reinforce notions that music-making is a White male pursuit and discourage others from participation later in life (Citron, 1993; Griffiths, 2020; McClary, 1991). Therefore, while this review focused predominantly at the individual rather than social level, it is important to note that access to music may be restricted and that music-making operates within a value-laden social context.

As our findings demonstrate that context and individual needs are important in determining the positive effects of music, it is likely that individual attributes also play a role. Indeed, it is widely accepted that personal traits have a significant impact on life experiences (Cole, 2009). However, as none of the articles that met the inclusion criteria explicitly considered the impact of intersectionality, this was an area that was not explored within this meta-ethnography. While Bailey and Davidson’s study [5] considered the differences between disadvantaged and middle-class music-makers, acknowledging that the benefits of music may vary according to an individual’s context, as with the majority of the synthesized studies the specific attributes of the participants within the populations studied were not outlined. Furthermore, although a few studies included demographical information such as the ethnicities or sexual orientations of their wider sample, the characteristics of the individual participants were not listed [26, 45]. As a result, this review is unable to explore how specific subgroups may use music to support their mental well-being. Despite this, our interpretation successfully highlights the pathways through which music supports positive mental well-being for participants in general, aiming to build a whole out of parts (Brookfield et al., 2019) and utilizing the accounts of participants from a range of age groups, nationalities, and genders. Our proposed model (Figure 1) could be drawn upon as a framework for subsequent studies that investigate how music can support the mental well-being of specific groups of people.

Notwithstanding these limitations, this meta-ethnography is the first to scrutinize the question of how participatory music engagement supports positive mental well-being. Through the analysis of 46 articles representing more than 2,000 participants, it proposes that participatory music supports mental well-being through four pathways that meet individual needs: managing and expressing emotions, facilitating self-development, providing respite, and facilitating connections. Given that music will always be a complex intervention, synthesis work such as this is arguably an essential way forward in elevating the impact of necessarily idiosyncratic qualitative studies. Indeed, for academics, the pathways presented here focus attention for further research in the field, providing the basis for studies that test hypotheses growing from this work or that aim to further understand the nuances and interplay between the different pathways. For music and health practitioners, the pathways can guide the development and delivery of future interventions to make use of the existing evidence base to support mental well-being.

## Supplemental Material

sj-pdf-1-qhr-10.1177_1049732320944142 – Supplemental material for How Participatory Music Engagement Supports Mental Well-being: A Meta-Ethnography Supplemental material, sj-pdf-1-qhr-10.1177_1049732320944142 for How Participatory Music Engagement Supports Mental Well-being: A Meta-Ethnography by Rosie Perkins, Adele Mason-Bertrand, Daisy Fancourt, Louise Baxter and Aaron Williamon in Qualitative Health Research

sj-pdf-2-qhr-10.1177_1049732320944142 – Supplemental material for How Participatory Music Engagement Supports Mental Well-being: A Meta-Ethnography Supplemental material, sj-pdf-2-qhr-10.1177_1049732320944142 for How Participatory Music Engagement Supports Mental Well-being: A Meta-Ethnography by Rosie Perkins, Adele Mason-Bertrand, Daisy Fancourt, Louise Baxter and Aaron Williamon in Qualitative Health Research

sj-pdf-3-qhr-10.1177_1049732320944142 – Supplemental material for How Participatory Music Engagement Supports Mental Well-being: A Meta-Ethnography Supplemental material, sj-pdf-3-qhr-10.1177_1049732320944142 for How Participatory Music Engagement Supports Mental Well-being: A Meta-Ethnography by Rosie Perkins, Adele Mason-Bertrand, Daisy Fancourt, Louise Baxter and Aaron Williamon in Qualitative Health Research
