# Supplementary figures and images for: How Participatory Music Engagement Supports Mental Well-being: A Meta-Ethnography
Source: Qual Health Res. 2020 Aug 5;30(12):1924–40. doi: 10.1177/1049732320944142 (PMC7502980; doi:10.1177/1049732320944142)

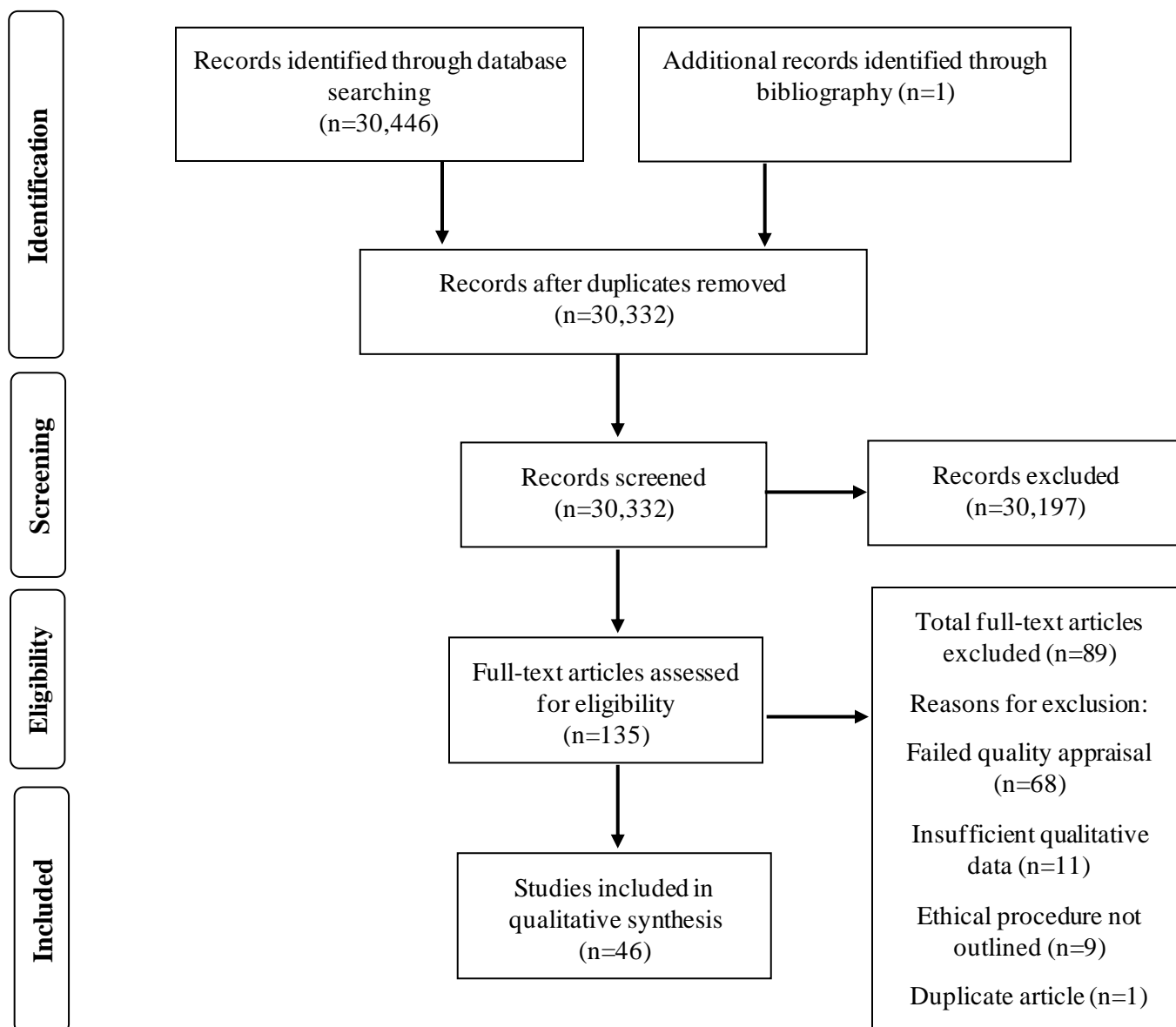

Supplementary Figure 1. Article selection flow diagram

Supplement: sj-pdf-1-qhr-10.1177_1049732320944142 – Supplemental material for How Participatory Music Engagement Supports Mental Well-being: A Meta-Ethnography [file sj-pdf-1-qhr-10.1177_1049732320944142.pdf]
