# Supplementary material for: How Participatory Music Engagement Supports Mental Well-being: A Meta-Ethnography
Source: Qual Health Res. 2020 Aug 5;30(12):1924–40. doi: 10.1177/1049732320944142 (PMC7502980; doi:10.1177/1049732320944142)
Supplement: sj-pdf-2-qhr-10.1177_1049732320944142 – Supplemental material for How Participatory Music Engagement Supports Mental Well-being: A Meta-Ethnography [file sj-pdf-2-qhr-10.1177_1049732320944142.pdf]

Supplementary Table 2. Summary of articles included in the review. Participant details and sample sizes refer to the qualitative components of studies only; for studies which employed multi-strategy designs the information relating to quantitative data is not included as it falls outside of the scope of this review.

**Citation**	**Objective**	**Country(s)**	**Type of music participation**	**Method(s)**	**Participants**	**Sample size**	**Analysis method**	**Key findings**
Ahmadi, F. (2011). Song lyrics and the alteration of self-image. *Nordic Journal of Music Therapy*, *20*(3), 225–241. https://doi.org/10.1080/08098131.2010.522718 [1]	To examine, from a patient perspective, the role music plays as a coping method when facing cancer	Sweden	Singing<br><br>*NB – music-listening is also included in the article, but only data relating to singing were extracted*	Semi-structured interviews	Cancer patients who used music during and after their illness. Mixed sex. Aged 24-73 years	1 participant explicitly engaged in singing<br><br>*NB – this participant is also included in article [2]*	Coding drawing on methods of grounded theory	The lyrics of religious, cheerful, and hard and heavy music can help cancer patients obtain a balance in their inner feelings by identifying with the person to whom the song lyrics are addressed
Ahmadi, F. (2013). Music as a method for coping with cancer: A qualitative study among Cancer patients in Sweden. *Arts & Health*, *5*(2), 152–165. https://doi.org/10.1080/17533015.2013.780087 [2]	To investigate patients' understandings of the role of music in coping and influencing wellbeing	Sweden	Singing<br><br>*NB – music-listening is also included in the article, but only data relating to singing were extracted*	Semi-structured interviews	Cancer patients who used music during and after their illness. Mixed sex. Aged 24-73 years	1 participant explicitly engaged in singing<br><br>*NB – this participant is also included in article [1]*	Coding drawing on methods of grounded theory	The situations and characteristics of individuals determine which types of music can be used as a useful or harming coping strategy
Ascenso, S., Perkins, R., Atkins, L., Fancourt, D., & Williamon, A. (2018). Promoting well-being through group drumming with mental health service users and their carers. *International Journal of Qualitative Studies in Health and Well-being*, *13* (1484219), 1–15. https://doi.org/10.1080/17482631.2018.1484219 [3]	To explore the extent to which group drumming interventions result in positive wellbeing change for mental health service users and their carers	United Kingdom	Group drumming	Semi-Structured interviews and focus groups	30 adult mental health service users, 6 informal and formal carers, and 3 participants who were both mental health service users and carers. Mixed sex. Age not specified	39<br><br>*NB – these participants are also included in article [33]*	Interpretative phenomenological analysis (IPA)	Social, psychological, and emotional wellbeing was identified for both mental health service users and their carers as a result of the group drumming workshops

Bailey, B., & Davidson, J. (2002). Adaptive characteristics of group singing: Perception from members of a choir for homeless men. *Musicae Scientiae*, *6*(2), 221–256. https://doi.org/10.1177/102986490200600206 [4]	To determine if group singing was a factor in promoting adaptive behavior among homeless men	Canada	Group singing	Semi-structured interviews	Members of a choir for homeless men who were aged 45-62 years	7<br><br>*NB – these participants are also included in article [5]*	Data were re-read multiple times to discover emergent themes	Group singing positively influenced emotional, social, and cognitive processes relating to clinical-type benefits, benefits from audience-choir reciprocity, group process, and benefits related to mental engagement
Bailey, B., & Davidson, J. (2005). Effects of group singing and performance for marginalised and middle-class singers. *Psychology of Music*, *33*(3), 269–303. https://doi.org/10.1177/0305735605053734 [5]	To explore the effects of group singing and performance in a choir for homeless and marginalized individuals with little or no musical training and a choir for middle class singers with low to high levels of musical training	Canada	Choirs	Interviews and focus groups	Members of a choir for homeless men, members of a mixed-sex choir for vulnerable adults, members of a mixed-sex choir of middle class members of the general public.<br>Age not specified.	23<br><br>*NB – 7 of these participants are also included in article [4]*	Interpretative phenomenological analysis (IPA)	The emotional effects of group singing are the same regardless of socio-economic status or musical training, but the interpersonal and cognitive components differ
Batt-Rawden, K., & Andersen, S. (2020).'Singing has empowered, enchanted and enthralled me'-choirs for wellbeing? *Health Promotion International*, *35*(1), 140–150. https://doi.org/10.1093/heapro/day122 [6]	To explore how women's perception of their own health and wellbeing can be affected by singing in a choir and how choral singing can impact on social inclusion	Norway	Choirs	Semi-structured interviews	Female choir members who were members of the general public. Aged 21-75 years	19	Grounded theory	Choral singing affected women's perceptions of their wellbeing, being seen as joyous, essential for their survival, a route to social connection, increasing self-confidence and self-esteem, and promoting social inclusion

Carolan, M., Barry, M., Gamble, M., Turner, K., & Mascarenas, O. (2012). The Limerick lullaby project: An intervention to relieve prenatal stress. *Midwifery*, *28*(2), 173–180. https://doi.org/10.1016/j.midw.2010.12.006 [7]	To explore the impact of singing lullabies during pregnancy	United Kingdom	Group lullaby singing classes	Qualitative interviews	Pregnant women recruited from an antenatal class. Age not specified	6 *NB – these participants are also included in article [8]*	Data were re-read multiple times in order to identify themes	Learning to sing lullabies resulted in feelings of relaxation, a sense of connection to the unborn baby and other pregnant women, and provided a means of communication after the baby's birth
Carolan, M., Barry, M., Gamble, M., Turner, K., & Mascarenas, O. (2012). Experiences of pregnant women attending a lullaby programme in Limerick, Ireland: A qualitative study. *Midwifery*, *28*(3), 321–328. https://doi.org/10.1016/j.midw.2011.04.009 [8]	To explore women's experiences of singing lullabies in pregnancy and their understanding of possible benefits for them and their infants	United Kingdom	Group lullaby singing classes	Qualitative interviews	Pregnant women recruited from antenatal classes, who had given birth within approx. three months. Age not specified	6 *NB – these participants are also included in article [7]*	Thematic content analysis	The women felt that the lullaby project benefitted them and their babies; music enabled the articulation of emotions, was powerful and pleasurable, and facilitated infant development
Clift, S., & Hancox, G. (2010). The significance of choral singing for sustaining psychological wellbeing: Findings from a survey of choristers in England, Australia and Germany. *Music Performance Research*, *3*(1), 79–96. [9]	To provide an understanding of wellbeing and health and provide a theoretical model of the mechanisms linking singing and wellbeing	Australia, United Kingdom, and Germany	Choirs	Questionnaire	Choral singers made up of the general public. Age and sex not specified	1124	Thematic analysis	Singing improved moods, focused attention, controlled breathing, which was calming, provided social support, cognitive stimulation, and regular commitment that was motivating and prevented inactivity
Clift, S., & Morrison, I. (2011). Group singing fosters mental health and wellbeing: Findings from the East Kent 'singing for health network project'. *Mental Health and Social Inclusion*, *15*(2), 88–97. https://doi.org/10.1108/20428301111140930 [10]	To describe the development and evaluation of a community singing initiative with mental health service users and supporters	United Kingdom	Choirs	Questionnaire	Members of choirs for the general public. Mixed sex. Age not specified	42	Responses were sorted into categories based on the scores participants recorded for a wellness survey	Group singing can have substantial benefits in aiding the recovery of people with serious and enduring mental health problems

D'Ardenne, P. & Kiyendeye, M. (2015). An initial exploration of the therapeutic impact of music on genocide orphans in Rwanda. *British Journal of Guidance and Counselling*, *43*(5), 559–569. https://doi.org/10.1080/03069885.2014.954237 [11]	To investigate whether music had transformational meaning for participants	Rwanda	Guitar, piano, singing, aural awareness, and song writing classes	Focus groups	Individuals who had been orphaned by the genocide in Rwanda. Mixed sex. Aged 19-32 years	13	Thematic analysis	Music changed how individuals saw their past, provided individuals with safe space, and provided a personal resource to face the future
Dingle, G., Brander, C., Ballantyne, J., & Baker, F. (2012). 'To be heard': The social and mental health benefits of choir singing for disadvantaged adults. *Psychology of Music*, *41*(4), 405–421. https://doi.org/10.1177/0305735611430081 [12]	To explore the personal experiences of choir members with chronic mental health problems, physical disabilities, and intellectual disabilities in relation to their wellbeing	Australia	Choirs	Semi-structured interviews	Choir members with mental illness or intellectual or physical disabilities. Mixed sex. Aged 31-74 years	21	Interpretative phenomenological analysis (IPA)	Music resulted in personal impact (such as positive emotions) and functional outcomes (such as health benefits)
Fingleton, L., O'Connor, J., & Stynes, G. (2018). Going backstage: A psychoanalytically informed study with amateur musicians in adult mental health services. *Psychoanalytic Psychotherapy*, 32 (1), 4–18. https://doi.org/10.1080/02668734.2017.1382564 [13]	To explore how playing a musical instrument could sublimate stress	United Kingdom	Playing a musical instrument	Three unstructured interviews with each participant	Male participants from the general population. Aged 42-68 years	6	A psycho-analytically informed analysis approach; rereading transcripts and identifying themes	Music is a transitional object and a means of containing stress; music plays a narcissistic function

Gale, N., Enright, S., Lewis, I., & Van Deursen, R.(2012). A pilot investigation of quality of life and lung function following choral singing in cancer survivors and their carers. *Ecancer*, *6* (261), 13. https://doi.org/10.3332/ecancer.2012.261 [14]	To evaluate quality of life and lung function before and after three months of choral singing in cancer survivors and their carers	United Kingdom	Choirs	Semi-structured interviews	Choirs members for the general public. Mixed sex. Aged 50-69 years	10	Phenomenological analysis	Choirs provided a focus, making individuals feel uplifted with positive benefits for their confidence and self-esteem
Goodrich, A. (2013). Health musicing in a community orchestra. *International Journal of Community Music*, *6*(1), 45–63. https://doi.org/10.1386/ijcm.6.1.45_1 [15]	To explore how health musicing functions within an orchestra and how participants perceive their engagement and health musicing with the local medical community	USA	Orchestra	Interviews and observations	Orchestra members who were employed within health care and members of the general public. Mixed sex. Aged not specified	Not specified but at least 5	Coding	Music provided community engagement and was seen to be healing
Hall, T. Mullen, A., Plummer, J., Berry, S., & Clancy, R. (2019). Sound practice: Exploring the benefits of establishing a music group on an acute mental health inpatient unit. *International Journal of Mental Health Nursing*, *28*(3), 697–705. https://doi.org/10.1111/inm.12569 [16]	To explore the impact of a music group activity on an acute mental health inpatient unit	Australia	Group singing and the playing of instruments such as piano, guitars, and percussions	Focus groups with four sets of participants	Staff members at a mental health facility, inpatients who took part in the music programme, music group facilitators. Age and sex not specified	31 who took part in music participation	Thematic analysis	Music participation had positive effects on moods, relationships, engagement, social connectedness, inclusion, ward atmosphere, and noise/aggression

Hopper, M., Curtis, S., Hodge, S., & Simm, R. (2016). A qualitative study exploring the effects of attending a community pain service choir on wellbeing in people who experience chronic pain. *British Journal of Pain, 10*(3), 124-134. https://doi.org/10.1177/2049463716638368 [17]	To explore participants' perceptions of the impact of a service-user-led community pain choir on their psychological wellbeing, self efficacy, and relationships with their chronic pain	United Kingdom	Choirs	Semi-structured interviews	Choir members who experienced chronic pain. Mixed sex. Aged 44-79 years	7	Thematic analysis	Participation in the choir enhanced feelings of self-worth, improving interpersonal relationships and overall wellbeing
Joseph, J., & Southcott, D. (2015). Singing in La Voce Della Luna Italian women's choir in Melbourne, Australia. *International Journal of Music Education*, *33*(1), 91-102. https://doi.org/10.1177/0255761414546244 [18]	To explore engagement by older members of La Voce Della Luna, an Italian women's community choir based in Melbourne	Australia	Choirs	Observations, focus groups, interview	Members of a female choir whose members held Italian heritage. Aged 60- 80+ years	6<br><br>5 members of the choir took part in a focus group and the musical director of the choir was interviewed<br><br>*NB – these participants are also included in article [19]*	Interpretative phenomenological analysis (IPA)	Choir participation encouraged social connection, combatted isolation, and led to new opportunities which resulted in recognition

Joseph, D., & Southcott, J. (2018). Music participation for older people: Five choirs in Victoria Australia. *Research Studies in Music Education*, *40*(2), 176-190. https://doi.org/10.1177/1321103X18773096 [19]	To report on five phenomeno-logical case studies of community singing groups comprised of older people who are active in the community in Melbourne	Australia	Choirs	Focus groups	Older adults who were members of 5 different choirs. Some of the choirs were mixed-sex, others were not. Age range not specified	24<br><br>22 accounts from the focus group of choir members. 2 semi-structured interviews from 2 choir directors.<br><br>*NB – 6 of these participants are also included in article [18]*	Interpretative phenomenological analysis (IPA)	Singing offered older people social connection, a sense of wellbeing, and musical engagement. This resulted in social cohesion, positive ageing, a sense of personal and group fulfilment, community engagement, and resilience
Joseph, J., Page-Shipp, R., & Van Niekerk, C. (2018). Singing and spirituality in a South African male voice group. *International Journal of Community Music, 11*(1), 21–38. https://doi.org/10.1386/ijcm.11.1.21_1 [20]	To report on interviews from a project on spirituality, music, and wellbeing in a community	South Africa	Choirs	Interviews made up of both closed and open questions	Members of the general public who participated in choirs. Male. Aged 60-90 years	12	Interpretative phenomenological analysis (IPA)	The choir fulfilled personal and musical needs, with the music positively impacting on social engagement, enhancing quality of life
Judd, M., & Pooley, J. (2013). The psychological benefits of participating in group singing for members of the general public. *Psychology of Music*, *42*(2), 269–283. https://doi.org/10.1177/0305735612471237 [21]	To explore the potential psychological benefits of singing in a choir for members of the general public	Australia	Choirs	Interviews	Members of the general public who participated in group singing. Mixed sex. Aged 33-72 years	10	Thematic analysis based on an interpretive approach	Both group and individual benefits were identified such as group dynamics and psychological benefits. A number of mediating factors were also found to impact on group outcomes including past experiences and the type of choir

Lagace, M., Briand, C., Desrosiers, J., & Lariviere, N. (2016). A qualitative exploration of a community-based singing activity on the recovery process of people living with mental illness. *British Journal of Occupational Therapy*, *79*(3), 178–187. https://doi.org/10.1177%2F0308022615599171 [22]	To explore the benefits of a community-based singing activity in Montreal, Canada and to identify the components that potentially explain these benefits	Canada	Choirs	Group interview	Participants with diagnosed mental illness. Mixed sex. Aged 32-69 years	18	Content analysis with open, axial, and selective coding	Engagement enabled the rediscovery of identity, improved self-confidence and social skills, having physical and cognitive benefits. This was due to the choir's supportive environment which allowed emotions to be expressed and expectations placed upon individuals
Lee, J., Davidson, J., & Krause, A. (2016). Older people's motivations for participating in community singing in Australia. *International Journal of Community Music*, *9*(2), 191–206. https://doi.org/10.1386/ijcm.9.2.191_1 [23]	To investigate the motivations of older people who regularly attend community singing groups in Australia	Australia	Choirs	Focus groups	Members of 3 community singing groups for the general public. Sex not specified. Aged 32-95 years	64	Inductive thematic analysis	The participants experienced different motivation factors for attending choirs at different stages of their engagement in the groups. Factors included pleasure, achievement, and fellowship
Li, S., & Southcott, J. (2012). A place for singing: Active music engagement by older Chinese Australians. *International Journal of Community Music, 5*(1), 59–78. https://doi.org/10.1386/ijcm.5.1.59_1 [24]	To explore the benefits of engaging in community singing groups through the experiences of a group of older Chinese Australians still active in their local community	Australia	Choirs	A focus group and semi-structured interviews	Older adults who emigrated from China to Australia. Women. Aged 59-85 years	8	Interpretative phenomenological analysis (IPA)	The benefits identified were emotional wellbeing, connections with the past, shared interests, and mental and physical wellbeing

Li, J., & Southcott, J. (2018). "Something to live for": Weekly singing classes at a Chinese university for retirees. *International Journal of Community Music, 36*(2), 283–296. https://doi.org/10.1177/0255761417729548 [25]	To explore the understandings and experiences of a group of older Chinese people who participate in singing lessons at the Tangshan Older People University, China	China	Singing lessons	Semi-structured interviews and observations	Members of a singing class for older adults and music facilitators. Mixed sex. Aged mid 40s-78 years	15	Interpretative phenomenological analysis (IPA)	The participants sought music-learning experiences and saw benefits in their ongoing music education, including emotional physical and mental wellbeing
Liebowitz, M., Tucker, M., Frontz, M., & Mulholland, S. (2015). Participatory choral music as a means of engagement in a veterans' mental health and addiction treatment setting. *Arts & Health, 7*(2), 137–150. https://doi.org/10.1080/17533015.2014.999246 [26]	To investigate how participation in a music based performance and instruction programme influenced the sense of engagement experienced by participants at a residential setting for at-risk veterans	USA	Choirs	Semi-structured interviews	War veterans. Mixed sex. Age not specified	6	A constant comparative process of analysis based on grounded theory	Opportunities to connect with others through shared interests contributed to a sense of engagement, with the choir offering a diversion from pressing concerns and serving as a means of facilitating adjustment
Livesey, L., Morrison, I., Clift, S., & Camic, P. (2012). Benefits of choral singing for social and mental wellbeing: qualitative findings from a cross-national survey of choir members. *Journal of Public Mental Health, 11*(1), 10–26. https://doi.org/10.1108/17465721211207275 [27]	To explore the benefits of choral singing for mental wellbeing and health as perceived by a cross-national sample of amateur choral singers	Australia, United Kingdom, and Germany	Choirs	Open ended questionnaire	Members of the general public who belonged to a choir. Mixed gender. Specific ages not specified	169	Thematic analysis	Choral singing resulted in perceived positive social, emotional, and physical benefits, which were shared regardless of age, gender, nationality, or wellbeing status

McNaughton, A., Aldington, S., Williams, G., & Levack, W. (2016). Sing your lungs out: A qualitative study of a community singing group for people with chronic obstructive pulmonary disease (COPD). *BMJ Open*, *7*(1), 1–7. http://dx.doi.org/10.1136/bmjopen-2016-012521 [28]	To explore the ways in which participation in a community singing group contributed to the health and wellbeing of patients with chronic obstructive pulmonary disease	New Zealand	Choirs	Interviews and focus groups	Individuals with chronic obstructive pulmonary disease or interstitial lung disease. Mixed sex. Aged 51-91 years	21	Coding and constant comparative methods based on grounded theory	The singing group reported benefits to health and wellbeing, with choirs providing a safe space, connection, purpose and growth, and participation in a meaningful physical activity
Newman, G., Maggott, C., & Alexander, D. (2015). Group drumming as a burnout prevention initiative among staff members at a child and adolescent mental health care facility. *South African Journal of Psychology*, *45*(4), 439–451. https://doi.org/10.1177/0081246315581346 [29]	To identify the subjective experiences of staff members at a child and adolescent mental health unit, who participated in a drumming group aimed at reducing burnout	South Africa	A drumming group	Semi-structured questionnaire	Staff members at a child and adolescent mental health unit. Age and sex not specified	17	Thematic analysis	Drumming and recreational music-making groups may be used as a burnout reduction strategy, giving a sense of belonging. Drumming was relaxing, expressive, granted a sense of accomplishment, and improved participants' moods
Osman, S., Tischler, V., & Schneider, J. (2016). 'Singing for the brain': A qualitative study exploring the health and well-being benefits of singing for people with dementia and their carers. *Dementia*, *15*(6), 1326–1339. https://doi.org/10.1177/1471301214556291 [30]	To explore the impact of Singing for the Brain, an intervention based on group singing activities developed by The Alzheimer's Society for people with dementia and their carers	United Kingdom	Group singing activities	Semi-structured interviews	People with dementia and their careers. Mixed sex, age not specified	20	Thematic analysis	Singing promoted social inclusion, improvements in relationships, memory and mood, and helped individuals to accept and cope with dementia

Parker, E. (2017). A phenomenology of one Southeastern African American church choir. *Bulletin of the Council for Research in Music Education, 212* (4), 57–74. https://doi.org/10.5406/bulcouresmusedu.212.0057 [31]	To describe the long-term singing experiences for participants in one Southeastern African American church choir in the USA	USA	Choirs	Interviews and observations	Members of a church choir for the general public, a church minister of music, and the music director. Mixed sex. Aged 25-62 years	24	Phenomenological analysis	Choir participation led to feelings of personal growth and community
Perkins, R., & Williamon, A. (2014). Learning to make music in older adulthood: A mixed-methods exploration of impacts on wellbeing. *Psychology of Music, 42*, 550–567. https://doi.org/10.1177%2F0305735613483668 [32]	To explore the role that learning musical instruments can play in enhancing wellbeing in older adulthood	United Kingdom	Learning an instrument- drums, keyboard, recorders, and guitars	Questionnaire and semi-structured interviews	Older adults. Mixed sex. Aged between 50-74 years.	21	Interpretative phenomenological analysis (IPA)	Learning music enhanced subjective wellbeing through subjective experiences of pleasure, social interactions, musical engagement in day-to-day life, fulfilment of musical ambition, and self-satisfaction through musical progress
Perkins, R., Ascenso, S., Atkins, L., Fancourt, D., & Williamon, A. (2016). Making music for mental health: How group drumming mediates recovery. *Psychology of Well-Being, 6*(11), 1–17. https://doi.org/10.1186/s13612-016-0048-0 [33]	To elucidate the features of a programme of group drumming known to enable mental health recovery	United Kingdom	Group drumming	Semi-structured interviews and focus groups	30 adult mental health service users, 6 informal and formal carers, and 3 participants who were both mental health service users and carers. Mixed sex. Age not specified	39 *NB – these participants are also included in article [3]*	Interpretative phenomenological analysis (IPA)	Group drumming provided a creative and mutual learning space aiding mental health recovery

Perkins, R., Yorke, S., & Fancourt, D. (2018). How group singing facilitates recovery from the symptoms of postnatal depression: A comparative qualitative study. *BMC Psychology, 6*(41), 1–12. https://doi.org/10.1186/s40359-018-0253-0 [34]	To elucidate the mechanisms of a group singing intervention in order to account for its recovery properties	United Kingdom	Group singing	Semi-structured focus groups	Mothers who had experienced symptoms of postnatal depression. Women. Age not specified	54	Thematic analysis	Group singing provided an authentic, social, and multicultural creative experience, was a tool to calm babies, provided immersive 'me time' for mothers, facilitated a sense of achievement and identity and enhanced the perceived mother-infant bond
Plumb, L., & Stickley, T. (2017). Singing to promote mental health and well-being. *Mental Health Practice, 20*(8), 31–36. https://doi:10.7748/mhp.2017.e1182 [35]	To explore the possible health and wellbeing effects of a community-based choir which aims to promote mental health and wellbeing	United Kingdom	Choirs	Semi-structured interviews	Members of the general public who belonged to a choir which promoted positive mental-health. Mixed sex. Age not specified	10	Thematic analysis	Members reported social and health benefits, personal accomplishments, and enjoyment
Reagon, C., Gale, N., Enright, S., Mann, M., & van Deursen, R. (2016). A mixed-method systematic review to investigate the effect of group singing on health related quality of life. *Complementary Therapies in Medicine*, *27*, 1–11. https://doi.org/10.1016/j.ctim.2016.03.017 [36]	To explore the effects of participation in community choirs on health-related quality of life in individuals who have had cancer or have been affected by cancer	United Kingdom	Choirs	Questionnaire, interviews, and focus groups	Individuals who were bereaved through cancer, had a diagnosis of cancer, family members or carers of individuals with cancer, those with chronic health conditions, and health care professionals. Mixed sex. Aged 53-68 years	49	Systematic analysis using coding	Participants saw the choirs as both an uplifting musical activity and a supportive community group. The choirs provided a spectrum of support options to meet the different needs and preferences of people affected by cancer

Rohwer, D., & Rohwer, M. (2012). How participants envision community music in Welsh men's choirs. *Research & Issues in Music Education, 10*(1), 1–15. [37]	To describe choristers' perceptions of Welsh men's choir participation	United Kingdom	Choirs	Focus groups	Members of a male choir for the general population. Aged 32-89 years	24	The content areas were described, with respondent trends and quotes being used to highlight each area	Musical, social, and nationalistic benefits were perceived
Rudd, E. (2013). Can music serve as a "cultural immunogen"? An explorative study. *International Journal of Qualitative Studies on Health and Well-being, 8*(1), 1–12. https://doi:10.3402/qhw.v8i0.20597 [38]	To explore how people in contemporary society may apply music in their everyday life to improve their health and wellbeing	Norway	Singing, piano, playing in a band, and song-writing<br><br>*NB – music-listening is also included in the article, but only data relating to singing were extracted*	Interviews	Members of the general population. Mixed sex. Age not specified	4 participants explicitly discuss active music-making	Open coding	Music was found to be a social and emotional resource, a supportive self object. and locus of control
Sapouna, L., & Pamer, E. (2014). The transformative potential of the arts in mental health recovery – an Irish research project. *Arts & Health, 8*(1), 1–12. https://doi.org/10.1080/17533015.2014.957329 [39]	To investigate the experience of arts participation for mental health service users in Cork, Ireland, and the potential of integrating the arts into mental health care	United Kingdom	Music and animation workshops featuring singing, drumming, and playing other instruments	Participatory observation, focus groups, and interviews	Service users with mental illnesses, music facilitators, and mental health staff. Age and sex not specified	47	An interpretive framework was employed	The arts created environments conducive to recovery through empowerment, connection-making, confidence-building, hope, story-telling, and story-making

Shakespeare, T., & Whieldon, A. (2017). Sing your heart out: community singing as part of mental health recovery, *Medical Humanites*, *44*(3), 153–157. http://dx.doi.org/10.1136/medhum-2017-011195 [40]	To evaluate the effectiveness of the Sing Your Heart Out project and to identify the key features which made the project distinctive	United Kingdom	Singing workshops	Interviews and focus groups	Members of singing workshops for those with mental health conditions and the general public, organizers, and music facilitators. Age and sex not specified	26	Thematic coding	Singing and the social workshops were beneficial for recovery, alongside the lack of pressure to discuss their conditions. The group was social and promoted belonging and structure
Skingley, A., Clift, S., Hurley, S., Price, S., & Stephens, L. (2018). Community singing groups for people with chronic obstructive pulmonary disease: Participant perspectives. *Perspectives in Public Health*, *138*(1), 66–75. https://doi.org/10.1177/1757913917740930 [41]	To investigate the views of participants with chronic obstructive pulmonary disease taking part in a singing for better breathing programme	United Kingdom	A singing programme with a focus on breathing	Interviews	Individuals who had chronic obstructive pulmonary disease or expressed problems breathing	37	Coding	Singing was a means to deflect attention away from breathing problems, it was seen as fun, and provided friendship and a positive emotions which led to motivation to participate in further activities
Southcott, J., & Nethsinghe, R. (2019). Resilient senior Russian-Australian voices: "We live to sing and sing to live". *Applied Research in Quality of Life*, *14*(1), 39–58. https://doi.org/10.1007/s11482-017-9580-1 [42]	To examine the understandings and meanings of shared music-making held by the members of the Young Hearts Russian choir in Australia and its impact on quality of life	Australia	Choirs	Semi structured interviews and focus groups	First generation Russian migrants to Australia. Mixed sex. Aged 50+ years	28	Interpretative phenomenological analysis (IPA)	Choir participation allowed individuals to maintain independence and resilience, enhancing quality of life by combatting social isolation

Unadkat, S., Clin, D., Camic, P., & Vella-Burrows, T. (2015). Understanding the experience of group singing for couples where one partner has a diagnosis of dementia. *The Gerontologist*, *57*(3), 469–478. https://doi.org/10.1093/geront/gnv698 [43]	To understand how group singing benefits people with dementia and their partners	United Kingdom	Singing groups	Semi-structured interviews	Couples, where one member had dementia. Mixed sex. Aged 61-89 years	34	Grounded theory	Group singing was joyful and accessible, encouraging participation in new experiences
Varvarigou, M., Hallam, S., Creech, A., & McQueen, H. (2012). Benefits experienced by older people who participated in group music-making activities. *Journal of Applied Arts and Health*, *3*(2), 183–198. https://doi.org/10.1386/jaah.3.2.183_1 [44]	To report on the benefits of participation in musical activities identified by people over the age of fifty, who participated in the Music for Life Project	United Kingdom	Singing, playing instruments including ukulele, steel pans, and percussion, and music appreciation	Questionnaire and interviews	Older adults. Mixed sex. Aged 50-93 years	27	Thematic analysis	Active engagement with music was reported to improve quality of life seen to offer cognitive, social, emotional, and physical benefits
Von Lob, G., Camic, P., & Clift, S. (2010). The use of singing in a group as a response to adverse life events. *International Journal of Mental Health Promotion*, *12*(3), 45–53. https://doi.org/10.1080/14623730.2010.9721818 [45]	To understand how individuals use community group singing as a response to adverse life events and to develop an explanatory framework of the social and psychological factors involved in this activity	United Kingdom	Community singing groups	Interviews	Members of a singing group who experienced an adverse life event in the past 10 years. Mixed sex. Aged 24-77 years	16	Interpretive phenomenological analysis (IPA)	Singing enhanced wellbeing as it helped individuals to cope or adapt to negative life events by being self-protective, self-restorative, and transformative. Music restored continuity with the past, was a vehicle for personal transformation, generated optimism about the future, and was distracting.

Warran, K., Fancourt, D., & Wiseman, T. (2019). How does the process of group singing impact on people affected by cancer? A grounded theory study. *BMJ Open, 9*(1), e023261. http://dx.doi.org/10.1136/bmjopen-2018-023261 [46]	To build an understanding of how the process of singing impacts on those who are affected by cancer, including patients, staff, carers, and those who have been bereaved	United Kingdom	Choirs	Focus groups and interviews	Patients with cancer, staff, carers, and bereaved who had participated for a minimum of 6 weeks in one of two choirs for people affected by cancer. Mixed sex. Age not specified	32	A systematic iterative approach informed by grounded theory	Choir participation helped to build resilience, provided social support, had psychological benefits including improving mood, was seen as fun, was holistic, and helped to build identities. Inspirational leaders, the challenge of learning music, and the uplifting songs also contributed to wellbeing
