# Supplementary material for: How Participatory Music Engagement Supports Mental Well-being: A Meta-Ethnography
Source: Qual Health Res. 2020 Aug 5;30(12):1924–40. doi: 10.1177/1049732320944142 (PMC7502980; doi:10.1177/1049732320944142)
Supplement: sj-pdf-3-qhr-10.1177_1049732320944142 – Supplemental material for How Participatory Music Engagement Supports Mental Well-being: A Meta-Ethnography [file sj-pdf-3-qhr-10.1177_1049732320944142.pdf]

Supplementary Table 1. Search terms.

**Music engagement**	**AND**	**Mental wellbeing**	**AND**	**Qualitative data**
Music OR		Wellness OR		Subjective Well Being OR
Music-Making OR Sing		Wellbeing OR		Subjective Well-Being OR
OR		Well-Being OR		SWB OR
Singing OR		Mental Health OR		Subjective OR
Song Writing OR Song-		Eudaimonic OR		Perspective OR
Writing OR Choirs OR		Eudaimonia OR		Perspectives OR
Orchestras OR		Eudaemonic OR		Standpoint OR
Music Groups		Eudaemonia OR		Standpoints OR
		Eudemonic OR		Point of View OR
		Eudemonia OR		Qualitative OR
		Hedonic OR		Interview OR
		Hedonism OR		Interviews OR
		Hedonistic OR		Focus Group OR
		Social OR		Observation OR
		Mood OR		Outlook OR
		Benefit OR		Viewpoint OR
		Stress OR		Stance OR
		Mental Illness OR		Attitude OR
		Depression OR		Understanding OR
		Anxiety OR		Conscious OR
		Patient		Themes OR
				Grounded
